# A New Non-Obese Steatohepatitis Mouse Model with Cardiac Dysfunction Induced by Addition of Ethanol to a High-Fat/High-Cholesterol Diet

**DOI:** 10.3390/biology13020091

**Published:** 2024-02-01

**Authors:** Seiji Shiraishi, Jinyao Liu, Yuki Saito, Yumiko Oba, Yuiko Nishihara, Satomichi Yoshimura

**Affiliations:** 1 Exploratory Research Department, EA Pharma Co., Ltd., Fujisawa-shi 251-8555, Kanagawa, Japan; seiji_shiraishi@eapharma.co.jp (S.S.); yuki.saitou.4az@asv.ajinomoto.com (Y.S.); yuiko_nishihara@eapharma.co.jp (Y.N.); 2 Student Medical Academia Investigation Lab, Yamaguchi University Graduate School of Medicine, Ube 755-8505, Yamaguchi, Japan; i019eb@yamaguchi-u.ac.jp (Y.O.); yoshi7ns@yamaguchi-u.ac.jp (S.Y.)

**Keywords:** ethanol, STHD-01, sympathetic activation, non-obese metabolic dysfunction-associated steatotic liver disease, cardiac dysfunction

## Abstract

**Simple Summary:**

Non-obese metabolic dysfunction-associated steatotic liver disease (MASLD) has been associated with cardiovascular-related mortality, leading to a higher mortality rate compared the general population. However, few reports have examined cardiovascular events in non-obese MASLD mouse models. Through the present study, we highlight a non-obese steatohepatitis mouse model with cardiac dysfunction produced using co-diet ethanol added to a high-fat/high-cholesterol diet (STHD-01) for 6 or 12 weeks. Incorporating ethanol into the STHD-01 diet regimen intensifies liver issues, such as inflammation and fibrosis, as well as cardiac dysfunction, potentially due to enhanced sympathetic nervous system activity. Alcohol, even when completely metabolized on the day of drinking, is a factor that exacerbates the progression of non-obese MASLD and cardiac dysfunction.

**Abstract:**

Non-obese metabolic dysfunction-associated steatotic liver disease (MASLD) has been associated with cardiovascular-related mortality, leading to a higher mortality rate compared to the general population. However, few reports have examined cardiovascular events in non-obese MASLD mouse models. In this study we created a mouse model to mimic this condition. In this study involving seven-week-old C57BL/6J male mice, two dietary conditions were tested: a standard high-fat/high-cholesterol diet (STHD-01) and a combined diet of STHD-01 and ethanol. Over periods of 6 and 12 weeks, we analyzed the effects on liver and cardiac tissues using various staining techniques and PCR. Echocardiography and blood tests were also performed to assess cardiac function and liver damage. The results showed that mice on the ethanol-supplemented STHD-01 diet developed signs of steatohepatitis and cardiac dysfunction, along with increased sympathetic activity, as early as 6 weeks. At 12 weeks, more pronounced exacerbations accompanied with cardiac dilation, advanced liver fibrosis, and activated myocardial fibrosis with sympathetic activation were observed. This mouse model effectively replicated non-obese MASLD and cardiac dysfunction over a 12-week period using a combined diet of STHD-01 and ethanol. This dietary approach highlighted that both liver inflammation and fibrosis, as well as cardiac dysfunction, could be significantly worsened due to the activation of the sympathetic nervous system. Our results indicate that alcohol, even when completely metabolized on the day of drinking, exacerbates the progression of non-obese MASLD and cardiac dysfunction.

## 1. Introduction

Metabolic dysfunction-associated steatotic liver disease (MASLD) can develop into metabolic dysfunction-associated steatohepatitis (MASH), leading to fibrosis, cirrhosis, and even hepatocellular carcinoma. It is a condition that affects roughly 25% of the global adult population, presenting significant health and economic challenges [1,2]. While MASLD is often linked to obesity, diabetes, and metabolic syndrome [3], there are cases of normal-weight individuals with MASLD, termed ‘non-obese MASLD’ or ‘lean MASLD’ [4,5]. Meta-analyses have reported that 19.2% of subjects in MASLD populations were lean and 40.8% were non-obese, and that the global prevalence of lean MASLD was 4.1% [6,7]. Patients with non-obese MASLD can exhibit the full range of MASH histopathological features, such as steatosis, lobular inflammation, hepatocyte ballooning, and fibrosis [8]. Despite similarities in pathological mechanisms with obese MASLD, like free fatty acid accumulation and liver insulin resistance [9,10], non-obese MASLD patients face a higher 15-year cumulative all-cause mortality rate (51.7%) compared to those with obese MASLD (27.2%) and without MASLD (20.7%), as indicated by a meta-analysis [5]. However, the unique features underlying non-obese MASLD are still not fully understood, and the prevalence of cardiac dysfunction (heart failure) is rapidly increasing in many parts of the world [11].

MASLD, a multisystem disease, impacts not only the liver but also other organs including the brain [12] and cardiovascular system [11,13]. This leads to an increased mortality rate compared to the general population [14]. Studies suggest that non-obese individuals with MASLD often have a healthier metabolic profile yet face similar risks of cardiovascular diseases and cancer as those with obese MASLD [15]. Specifically, lean MASLD patients show a 50% higher all-cause mortality rate and more than double the cardiovascular mortality compared to healthy lean individuals [16]. Efforts have been made to improve MASLD therapeutics and the survival of MASLD patients with cardiac dysfunction, but the rates of cardiac dysfunction mortality remain elevated [17,18], and an effective treatment for MASLD has not been established [19]. This is at least partly because the underlying mechanisms are not yet clear and the existing animal models are limited. There is currently no wild-type animal model that adequately mimics the MASLD state in humans, for both obese and non-obese individuals.

Medical practice categorizes fatty liver disease into alcoholic liver disease and MASLD, based on daily alcohol consumption limits for metabolization (20 g for women, 30 g for men). The influence of drinking patterns and dietary fats is significant in metabolic liver disease and incorporating ethanol into unbalanced diets is increasingly common, as highlighted by Boyle et al. in 2018 [19].

The influence of sympathetic nervous system dominance is significant in the progression of liver fibrosis [20,21]. Chronic alcohol consumption, even non-excessive alcohol use, correlates with sympathetic nervous system dominance, affecting liver fibrosis and cardiac function, as shown in rats in two previous studies and an apolipoprotein E/low-density lipoprotein receptor (ApoE/LDLR) double-knockout mice in another study [22,23,24]. However, it remains unclear whether there is an interaction between ethanol and MASH-induced diets in liver fibrosis and cardiac dysfunction progression.

In our prior study, we observed that mice on a STHD-01 diet (a high-fat diet designed to induce steatohepatitis, rich in saturated fats and cholesterol) developed steatohepatitis and fibrosis, but not obesity or diabetes [25]. Here, we investigated the combined effects of ethanol and STHD-01 on mice with steatohepatitis, fibrosis, and cardiac dysfunction. The underlying mechanisms, especially those involving hepatic and cardiac sympathetic activation, were examined.

## 2. Materials and Methods

### 2.1. Mouse Model and Diets

In the study, seven-week-old C57BL/6J male mice were assigned to six groups: mice fed a 6-week STHD-01 diet with ethanol (FUJIFILM Wako, Tokyo, Japan) (STHD-01, Et (+): n = 6) or without ethanol (STHD-01, Et (−): n = 6); mice fed a 6-week standard diet (SD) without ethanol (SD, Et (−): n = 7); mice fed a 12-week STHD-01 diet with ethanol (STHD-01, Et (+): n = 7) or without ethanol (STHD-01, Et (−): n = 6); and mice fed a 12-week SD without ethanol (SD, Et (−): n = 7).

As in the referenced study [25], the standard diet (SD) was based on the AIN-93G formula, while the STHD-01 diet comprised 40% fat and 5% cholesterol. To acclimate to ethanol, mice initially received a 5 g/dL ethanol–water solution for the first week, followed by a 10 g/dL solution for the remaining 5 or 11 weeks, totaling 6 or 12 weeks of treatment. Ethanol was offered separately from the STHD-01 diet, mixed with water in a drinking bottle. Both were available ad libitum. The study also involved monitoring the mice’s weight and their consumption of food and ethanol weekly.

### 2.2. Echocardiography Examination

At both 6 and 12 weeks, the mice were anesthetized and underwent transthoracic echocardiography, as in our previous study [24]. Measurements like the left ventricle (LV) end-diastolic (LVDd) and end-systolic diameters (LVSd) were taken. The LV fractional shortening (LVFS) and ejection fraction (LVEF) were calculated for cardiac function evaluation. These assessments adhered to guidelines set by the American Society of Echocardiography [26]. This approach allowed for a thorough analysis of the cardiac structural and functional changes under the experimental conditions.

### 2.3. Blood and Tissue Collection

Blood samples were taken from the LV of all mice. After perfusion with phosphate-buffered saline (PBS), liver and LV tissues were harvested under anesthesia. Some tissue samples were preserved in RNA*later* for RNA stabilization and stored at −80 °C. The remaining tissues were fixed in paraformaldehyde, treated with sucrose, and stored at 4 °C. These procedures align with those used in previous studies [21,27], ensuring consistency in the methodological approach.

### 2.4. Histopathological and Fluorescence Immunohistochemical Examinations

Liver and LV tissues were prepared and analyzed using hematoxylin and eosin (HE), Oil Red O, and Sirius Red staining techniques to assess histological changes. The stained samples were examined under a microscope (BZ-X800; Keyence, Osaka, Japan) with particular attention to the quantification of Oil Red O and Sirius Red stained areas. The analysis was based on samples from 5 visual fields at 20× magnification, ensuring thorough and representative data collection to evaluate fat deposition and fibrosis.

Immunofluorescence staining was conducted on liver and LV samples using specific primary antibodies, including CD68 (SCB sc-9139, a rabbit polyclonal antibody, 1:200) and tumor necrosis factor alpha (TNF-α; AF-410-NA, a goat polyclonal antibody, 1:200) for liver inflammation, and TH tyrosine hydroxylase (TH; OST00324W, a goat polyclonal antibody, 1:200) for liver and LV sympathetic activation. The samples underwent secondary antibody incubation, including Alexa Fluor^®^ 488 AffiniPure alpaca anti-rabbit IgG (#611-545-215, 1:500; Jackson ImmunoResearch Laboratories, West Grove, PA, USA) for CD68, and Cy3-AffiniPure donkey anti-goat IgG for TNF-α and TH (705-165-147, 1:500). Nucleus staining was conducted with TO-PRO^®^-3 iodide (TO-PRO-3; Molecular Probes, Eugene, OR, USA). The stained samples were examined under a confocal microscope (LSM5 Pascal/ver. 3.2; Carl Zeiss MicroImaging, Zeiss, Oberkochen, Germany), and the positive areas were quantified using an image analysis system (ImageJ, 2023). This analysis involved sampling from 15 visual fields at 63× magnification, ensuring a comprehensive evaluation of inflammation and sympathetic activation in the tissues.

### 2.5. Real-Time Reverse Transcriptase Polymerase Chain Reaction (RT-PCR)

Total RNA was extracted from the frozen liver and LV tissues using specific kits, including the RNeasy^®^ Mini Kit for liver and RNeasy^®^ Fibrous Tissue Midi Kit for LV (Qiagen, Tokyo, Japan). The process included gDNA removal and cDNA synthesis, following the manufacturer’s guidelines (ReverTra Ace^®^ qPCR RT Master Mix with gDNA Remover; Toyobo, Osaka, Japan). The RT-PCR was conducted with the Applied Biosystems StepOne™ system (Applied Biosystems, Foster City, CA, USA). For gene expression analyses, specific assays were used to measure the mRNA levels of genes such as *Cd68* (Assay ID: Mm03047340_m1), *Tnf-a* (Assay ID: Mm99999068_m1), collagen 1a1 (*Col 1a1*; Assay ID: Mm00801666_g1), neuropeptide Y (*Npy*; Assay ID: Mm00445771_m1), and glyceraldehyde-3-phosphate dehydrogenase (*Gapdh*; Assay ID: sMm99999915_g1). These expressions were normalized against *Gapdh* mRNA in the same cDNA sample, using a comparative quantitative method. The results are expressed as fold changes relative to the SD, Et (−) group at 6 weeks. This approach allows for a precise comparison of gene expression changes across different groups and time points.

### 2.6. Blood Chemistry Tests

Blood alanine aminotransferase (ALT), aspartate aminotransferase (AST), total glucose (GLU), total cholesterol (TCHO), high-density lipoprotein cholesterol (HDL-C), and triglyceride (TG) levels were measured using FDC4000i and DRI-CHEM NX600V IC automatic analyzers (FUJIFILM Corporation, Tokyo, Japan).

### 2.7. Determination of Hepatic TG and TCHO Levels

To determine hepatic TG and TCHO levels, samples were obtained from frozen livers using the Folch method, and then hepatic TG and TCHO levels (mg/g liver) were determined using a Triglyceride kit (Code No.: 432-40201; Wako Pure Chemical Industries, Osaka, Japan) and LabAssay Cholesterol kit (Code No.: 294-65801, Wako Pure Chemical Industries) according to the manufacturer’s instructions in all mice.

### 2.8. Analysis of the Blood Morning-Residual Ethanol

In this study, a specific calibration experiment determined the appropriate concentration of ethanol in water to use. Plasma samples were analyzed to measure residual ethanol levels using a specified saliva alcohol testing kit (QED A150; product code #31150, ToxTests, Dayton, MT, USA), following manufacturer guidelines.

The study defined lobular inflammation in steatohepatitis based on CD68 and TNF-α levels in liver sections and mRNA expressions. Advanced liver fibrosis, or bridging fibrosis, was identified through Sirius Red staining and mRNA expression analysis of collagen. This methodology aligns with previous research [28] and offers a detailed approach to diagnosing and understanding liver conditions in the study.

### 2.9. Statistical Analyses

Continuous data were presented as mean ± standard deviation. Outliers were identified and excluded using the Smirnov–Grubbs test. Group comparisons of continuous variables were conducted using one-way ANOVA, with post hoc testing for significant findings. The statistical significance was set at a *p* value of less than 0.05, and analyses were carried out using specific statistical software (OMS Publishing, Saitama, Japan). This approach ensured a rigorous and standardized evaluation of the data.

## 3. Results

### 3.1. Animal Characteristics

**Figure 1:**
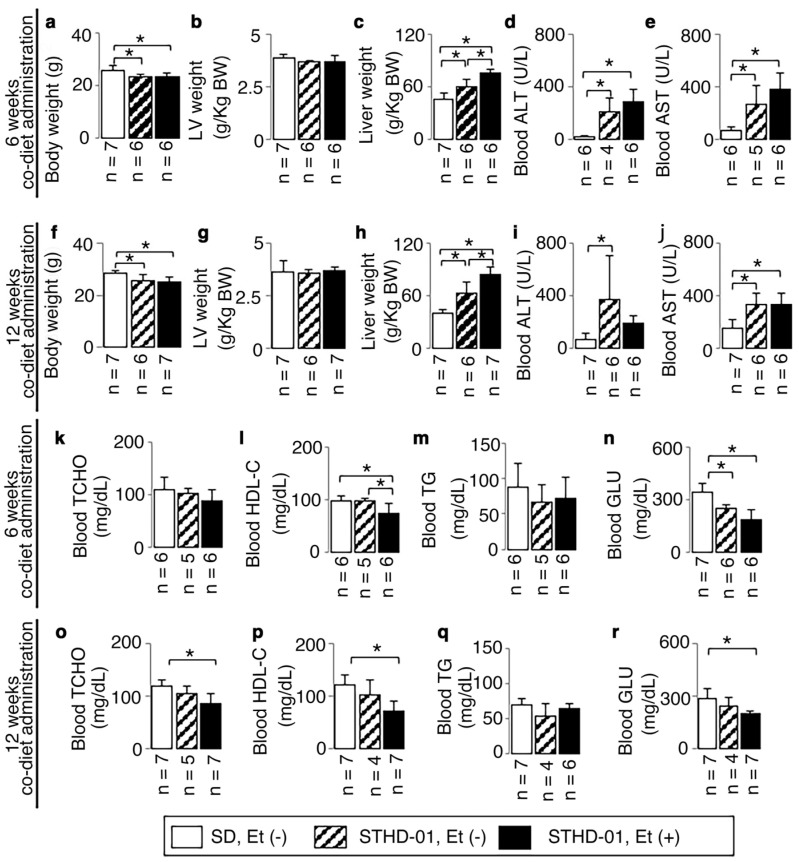
Basic animal characteristics. Representative results of body weight (BW) (**a**,**f**), LV weight (**b**,**g**), liver weight (**c**,**h**), blood ALT (**d**,**i**), blood AST (**e**,**j**), blood TCHO (**k**,**o**), HDL-C (**l**,**p**), TG (**m**,**q**), and GLU (**n**,**r**) at the end of 6 and 12 weeks of co-diet of STHD-01 and ethanol. Data are presented as the means ± SDs. * *p* < 0.05 using ANOVA followed by Bonferroni/Dunn post hoc test.

Body weights were decreased both in mice fed an STHD-01 with ethanol and those fed an STHD-01 without ethanol after 6 weeks, and the weight decreases were maintained at 12 weeks (Figure 1a,f).

There were no significant increases in LV weight (Figure 1b,g) in all mice. In contrast, a significant increase in liver weight was observed both in mice fed an STHD-01 with ethanol and those fed an STHD-01 without ethanol after 6 and 12 weeks (Figure 1c,h), accompanied by hepatic injury as shown by the increased blood ALT and AST from 6 weeks onwards (Figure 1d,e).

The co-diet of STHD-01 and ethanol produced low levels of blood HDL-C both after 6 and 12 weeks of administration (Figure 1i,p). No significant increase was observed in blood TCHO, TG, or GLU levels, but TCHO was significantly decreased at 12 weeks (Figure 1o) and GLU was significantly decreased both at 6 and 12 weeks (Figure 1n,r).

Morning-residual blood ethanol concentrations were 0.1083 ± 0.095 (0, 0.175, 0.15) and 0.0625 ± 0.125 (0.25, 0, 0, 0) mg/mL in mice fed a co-diet of SHTD-01 and ethanol for 6 and 12 weeks, suggesting that the ethanol was almost completely metabolized on the same day of drinking.

Based on the above results, a non-obese wild-type mouse model without hyperlipidemia and diabetes was created, and the ethanol consumption was set at a non-excessive level.

### 3.2. A Co-Diet of STHD-01 and Ethanol Produced Hepatic Steatosis, Inflammation, and Advanced Liver Fibrosis

**Figure 2:**
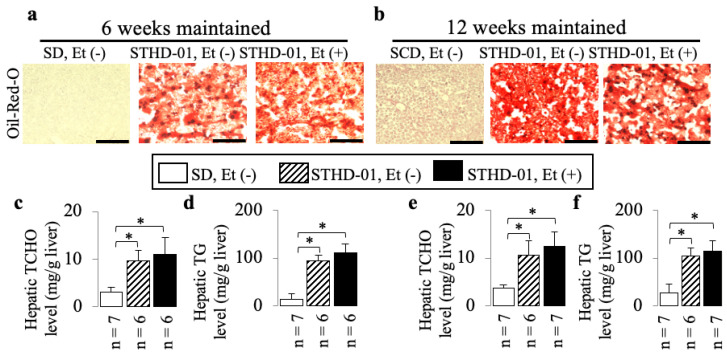
The additive effects of ethanol on the features of fatty liver in mice consuming STHD-01. Examples of Oil Red O-stained liver sections (**a**,**b**). Representative results of hepatic TCHO (**c**,**e**) and hepatic TG (**d**,**f**) levels at the end of 6 and 12 weeks of co-diet administration. Bar = 100 µm. Data are presented as the means ± SDs. * *p* < 0.05 using ANOVA followed by Bonferroni/Dunn post hoc test.

**Figure 3:**
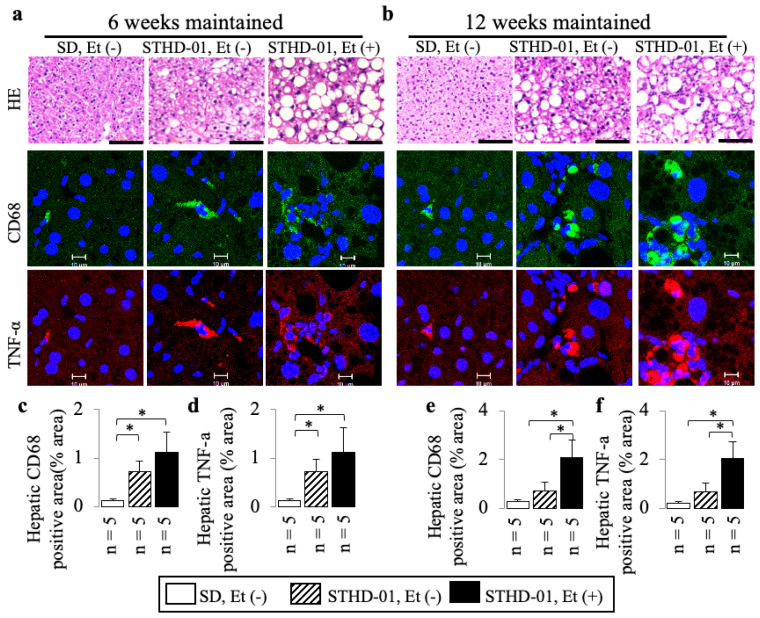
The additive effects of ethanol on steatohepatitis in mice consuming STHD-01. Examples of HE-stained liver sections (upper row), and CD68 (middle row) and TNF-α (lower row) immunostained liver sections (**a**,**b**). Bars = 100 μm for HE, but 10 μm for CD68 and TNF-α. Representative results of hepatic CD68-positive (**c**,**e**) and TNF-α-positive (**d**,**f**) areas at the end of 6 and 12 weeks of co-diet administration. Data are presented as the means ± SDs. * *p* < 0.05 using ANOVA followed by Bonferroni/Dunn post hoc test.

**Figure 4:**
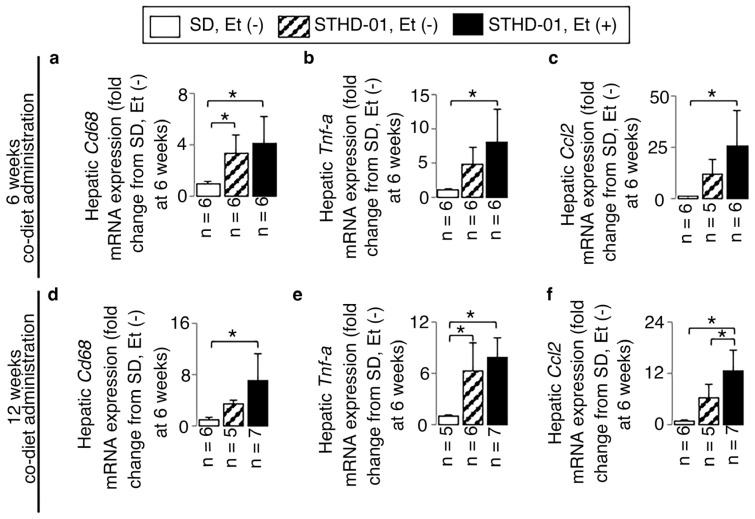
The additive effects of ethanol on the steatohepatitis-associated mRNA expressions in mice consuming STHD-01. Representative results of hepatic *Cd68* (**a**,**d**), *Tnf-α* (**b**,**e**), and *Ccl2* (**c**,**f**) mRNA expressions at the end of 6 and 12 weeks of co-diet administration. Data are presented as the means ± SDs. * *p* < 0.05 using ANOVA followed by Bonferroni/Dunn post hoc test.

**Figure 5:**
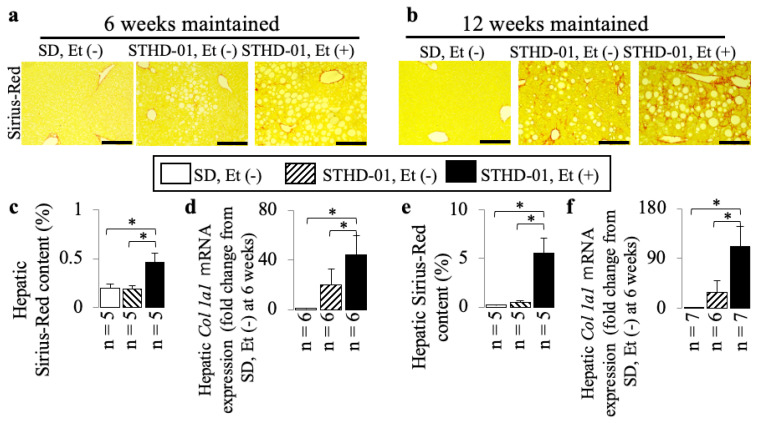
The additive effects of ethanol on advanced liver fibrosis in mice consuming STHD-01. Examples of Sirius Red-stained liver sections (**a**,**b**). Bars = 200 μm. Representative results of hepatic Sirius Red area (**c**,**e**) and hepatic *Col 1a1* mRNA expression (**d**,**f**) at 6 and 12 weeks of co-diet administration. Data are presented as the means ± SDs. * *p* < 0.05 using ANOVA followed by Bonferroni/Dunn post hoc test.

STHD-01 alone induced hepatic steatosis (Figure 2) and inflammation (Figure 3 and Figure 4) without fibrosis (Figure 5) at 6 weeks and 12 weeks; these effects were not observed in the mice maintained on the SD without ethanol.

A co-diet of STHD-01 and ethanol resulted in significant hepatic steatosis (Figure 2) and inflammation (Figure 3 and Figure 4) with fibrosis, but no bridging fibrosis was observed (Figure 5a) at 6 weeks. More pronounced exacerbations in inflammation and fibrosis, as shown by the remarkable lobular inflammation (Figure 3b and Figure 4d–f) and fibrosis (Figure 5b,e,f) with bridging fibrosis (Figure 5b), were noted at 12 weeks of administration. These effects were not observed in the mice maintained on the SD without ethanol.

Lobular inflammation was characterized using HE liver sections based on small nuclear aggregates and hepatocyte ballooning (Figure 3a,b) and increased levels of CD68 and TNF-α (Figure 3) in immunostained liver sections and on upregulated mRNA expressions of *Cd68*, *Tnf-α*, and *Ccl2* in RT-PCR (Figure 4). We observed 15.7- and 16.1-fold increases in the CD68- and TNF-α-positive areas accompanied by 4.1-, 8.1-, and 25.4-fold up-regulated *Cd68*, *Tnf-α*, and *Ccl2* mRNA expressions compared to those of mice fed an SD without ethanol at 6 weeks (all *p* < 0.05).

The study identified significant liver fibrosis at 12 weeks in mice fed the STHD-01 and ethanol diet. This advanced fibrosis was marked by bridging fibrosis, visible in Sirius Red-stained liver sections (Figure 5b), the increases in the Sirius Red-stained area (Figure 5e), and *Col 1a1* mRNA expressions (Figure 5f). Compared to mice on a standard diet without ethanol at 6 weeks, a 27.5-fold increase in the Sirius Red area along with an 86.3-fold increase in the *Col 1a1* mRNA expression at 12 weeks were observed, highlighting the diet’s impact on liver fibrosis development.

### 3.3. A Co-Diet of STHD-01 and Ethanol Caused Cardiac Dysfunction from 6 Weeks Onwards, and Cardiac Dilation with Myocardial Fibrosis at 12 Weeks of Administration

**Figure 6:**
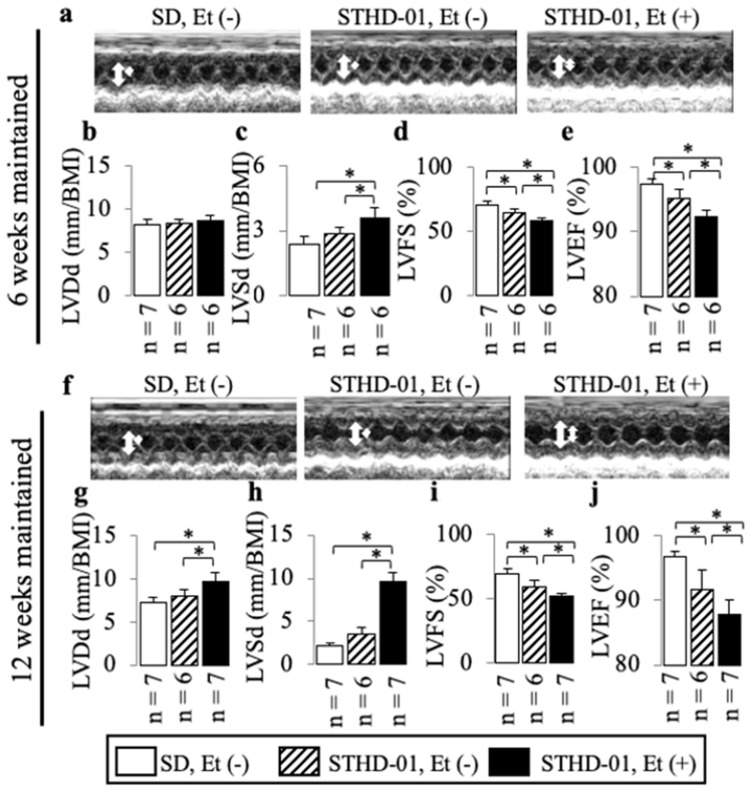
The additive effects of ethanol on cardiac dilation and dysfunction in mice consuming an STHD-01 diet. Examples of echocardiography (**a**,**f**). Representative results of LVDd (**b**,**g**), LVSd (**c**,**h**), LVFS (**d**,**i**), and LVEF (**e**,**j**) at the end of 6 and 12 weeks of co-diet administration; thick arrows represent LVDd, thin arrows represent LVSd. Data are presented as the means ± SDs. * *p* < 0.05 using ANOVA followed by Bonferroni/Dunn post hoc test.

**Figure 7:**
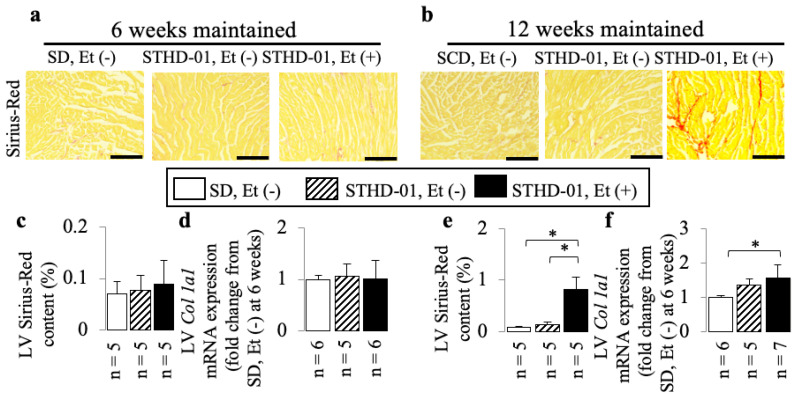
The additive effects of ethanol on myocardial fibrosis in mice consuming an STHD-01 diet. Examples of Sirius Red-stained LV sections (**a**,**b**). Representative results of Sirius Red area (**c**,**e**) and *col 1a1* mRNA expression (**d**,**f**) at the end of 6 and 12 weeks of co-diet administration. Bar = 200 µm. Data are presented as the means ± SDs. * *p* < 0.05 using ANOVA followed by Bonferroni/Dunn post hoc test.

In mice treated with both STHD-01 and ethanol, a higher incidence of cardiac dysfunction was observed from 6 weeks onwards, compared to STHD-01-fed mice, as shown by the increased LVSd (Figure 6a,c) and decreased LVFS (Figure 6d) and LVEF (Figure 6e), and at 12 weeks the co-diet-fed mice exhibited more pronounced exacerbations (Figure 6f,h–j) accompanied by cardiac dilation (as shown by the increased LVDd; Figure 6f,g), LV myocardial fibrosis (as shown by the increased Sirius Red area: Figure 7b,e), and up-regulation of *Col 1a1* mRNA expression (Figure 7f).

### 3.4. A Co-Diet of Ethanol and STHD-01 Induced Features of Hepatic and LV Myocardial Sympathetic Activation

**Figure 8:**
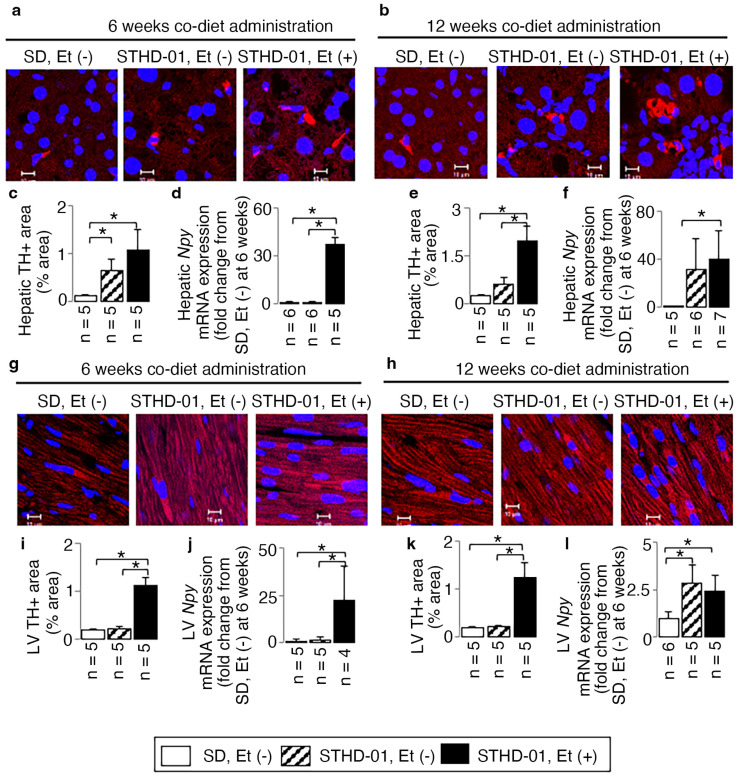
The additive effects of ethanol on the hepatic and cardiac sympathetic activation in mice consuming STHD-01. Examples of TH-immunostained liver (**a**,**b**) and LV sections (**g**,**h**), and representative results of hepatic (**c**,**e**) and LV (**i**,**k**) TH-positive areas, and hepatic (**d**,**f**) and LV (**j**,**l**) *Npy* mRNA expressions at the end of 6 and 12 weeks of co-diet administration. Bars = 10 μm. Data are presented as the means ± SDs. * *p* < 0.05 using ANOVA followed by Bonferroni/Dunn post hoc test.

Mice on a diet combining ethanol and STHD-01 showed increased sympathetic activation in both liver and LV tissue, as compared to those on the STHD-01, or standard diet, alone. This was evidenced by higher levels of the sympathetic marker TH in liver (Figure 8a–c,e) and LV (Figure 8g,i,k) tissue, and the up-regulation of the sympathetic activation marker (*Npy*) mRNA expression (Figure 8d,f for liver, Figure 8j,l for LV). There were 14- and 6.6-fold increases in TH-positive areas accompanied by 40.8- and 2.4-fold up-regulated *Npy* mRNA expressions for liver and LV tissue at 12 weeks of administration compared to those of the mice fed an SD without ethanol at 6 weeks (all *p* < 0.05); in addition, a 22-fold up-regulated LV *Npy* mRNA expression was observed at 6 weeks when compared with mice fed an SD without ethanol (*p* < 0.05).

## 4. Discussion

The first goal of the present study was to determine whether wild-type mice (C57BL/6J) on a co-diet of ethanol and STHD-01 would exhibit the features of steatohepatitis with advanced liver fibrosis and cardiac dysfunction observed in many MASLD patients. The main findings of this study were that a 12-week co-diet of ethanol and STHD-01 (1) exacerbated liver inflammation and advanced fibrosis (bridging fibrosis) compared with mice fed an STHD-01 alone, (2) led to the development of cardiac dysfunction as well as LV myocardial fibrosis without obesity, hyperglycemia, or hyperlipidemia, and (3) induced liver and LV sympathetic activation accompanied by hepatic lobular inflammation with bridging fibrosis as well as LV dilation with myocardial fibrosis.

Cardiovascular disease and malignancies are the two leading causes of death in patients with MASLD [29,30]. Efforts have been made to improve the survival of MASLD patients with cardiac dysfunction and to develop MASLD therapeutics, but the rates of cardiac dysfunction mortality remain elevated [13], and an effective treatment for MASLD has not been established [30]. This study highlights the challenge of finding an animal model that accurately represents human diseases. The results from this study differ from those using other mouse [31] and rat [23] models, which showed less hepatic inflammation and no fibrosis when fed high-fat or ethanol-liquid diets. This indicates a unique aspect of the model used in this study, emphasizing its potential usefulness in understanding human disease mechanisms more closely. Previous research on obese ApoE/LDLR double knockout mice with hyperlipidemia showed steatohepatitis and bridging fibrosis after a low-carbohydrate–high-protein–high-fat diet and ethanol regimen [32]. However, this was in older, 28-week-old mice. The current study suggests that a 12-week STHD-01 and ethanol co-diet (even though the ethanol was almost completely metabolized on the day of drinking) can effectively model non-obese steatohepatitis with advanced liver fibrosis and cardiac changes in younger mice, mirroring some conditions seen in non-obese MASLD patients. This indicates the potential of this model to represent certain human disease states more closely.

Around 70% of the global adult population drinks alcohol, and much emphasis has been placed on public health challenges to reduce excessive alcohol consumption [19]. However, metabolic risk factors, i.e., a nutritionally unbalanced dietary intake with some level of alcohol consumption now coexist in a large segment of the population [19]. Recent cross-sectional studies have shown that non-heavy alcohol use is associated with the development of liver fibrosis and MASH [33,34]. Therefore, it is important to examine the combined effects of alcohol consumption and metabolic risk factors, such as excessive calorie intake and unbalanced diets. These factors may interact and influence the progression of liver disease. In this study, an STHD-01 containing 5% cholesterol and 40% fat induced a state of steatohepatitis in a short amount of time, unlike another high-fat/high-cholesterol solid diet (0.2% cholesterol with 30% fat) (see the 2016 review of Ejima et al. [25]). Our present investigation showed that mice fed a co-diet of STHD-01 and ethanol exhibited higher rates of LV myocardial fibrosis with cardiac dysfunction onset with steatohepatitis compared to those fed an STHD-01 alone. Moreover, mice fed a co-diet of ethanol with STHD-01 produced significant bridge fibrosis in the liver; these effects were not observed in the mice maintained on the STHD-01 alone, which indicated that the ethanol aggravated liver fibrosis in mice fed an STHD-01. Liver fibrogenesis post-alcohol intake is influenced by acetaldehyde, ethanol’s primary metabolite during detoxification. Acetaldehyde contributes to collagen I transcription and indirectly stimulates TGF-β1 synthesis, both crucial in fibrogenic processes [35]. The present results demonstrated that a 12-week co-diet of STHD-01 and ethanol, even without morning-residual ethanol in blood, induced a remarkable increase in the hepatic Sirius Red area, observed using Sirius Red staining, and a remarkable upregulation of the *Col 1a1* mRNA expressions in the wild-type mice. Furthermore, because acetaldehyde is mainly metabolized by a mitochondria-localized enzyme (acetaldehyde dehydrogenase) in the liver, and the high levels of cholesterol contained in STHD-01 are also metabolized to bile acids in the same liver mitochondria, a co-diet of STHD-01 and alcohol may overburden mitochondria, preventing detoxification, and eventually exacerbating hepatocyte injury, inflammation, and fibrosis. This study suggests that even in cases where alcohol is fully metabolized on the same day, its consumption, especially in conjunction with a diet high in calories and nutritionally imbalanced, can exacerbate steatohepatitis and fibrosis in patients with metabolic risk factors. Therefore, avoiding alcohol could improve outcomes in MASLD patients.

MASLD is linked to early changes in cardiac metabolism, leading to functional and structural myocardial alterations and a heightened risk of cardiac dysfunction. While the exact mechanisms remain unclear, research, including Tsutsumi et al.’s 2021 review, suggests that MASLD patients are more likely to suffer from cardiac events than liver disease. Inflammatory hepatic lesions are believed to play a key role in the development of cardiac complications associated with MASLD [13]. Lean individuals with MASLD face a significantly higher risk of mortality compared to their healthy counterparts, with a 50% increase in all-cause mortality and more than double the rate of cardiovascular deaths [16]. This underscores the severe impact of MASLD even in patients without obesity. However, the complex pathophysiology underlying the initiation and progression of steatohepatitis with advanced liver fibrosis is incompletely understood [36]. Our present investigation showed that LV myocardial fibrosis with cardiac dysfunction occurred simultaneously with steatohepatitis with advanced fibrosis in 19-week-old male wild-type mice after 12 weeks of feeding with a co-diet of STHD-01 and ethanol. The underlying mechanisms—especially hepatic sympathetic activation-associated inflammatory hepatic lesions with advanced fibrosis and cardiac dysfunction with myocardial fibrosis—were the main focuses of the present study.

The sympathetic nervous system plays a crucial role in protecting body tissues from environmental and internal challenges. It modulates various processes such as inflammation, nociception, and immune system responses [37]. Research indicates that the sympathetic nervous system can influence gastrointestinal inflammation [38] and liver fibrosis through neurotransmitters like norepinephrine [20]. Additionally, a study in rats showed that circadian disruption could exacerbate cardiac remodeling post-myocardial infarction, leading to worsened cardiac dysfunction, LV dilation, and increased cardiac fibrosis, likely due to sympathetic nervous system activation. The suppression of sympathetic activity attenuated circadian disruption-related cardiac dysfunction [39]. In this study, ethanol consumption was shown to induce hepatic sympathetic activation in wild-type mice fed the STHD-01 diet. This was evidenced by increased levels of the sympathetic activation marker TH in liver sections and elevated *Npy* mRNA expression, as demonstrated with RT-PCR. The effects were noticeable from 6 weeks and became more pronounced by 12 weeks. Increased cardiac sympathetic activity was observed at the same time points. It is well known that obesity, hyperglycemia, and hyperlipidemia are risk factors in cardiac dysfunction. The study concludes that a combined diet of STHD-01 and ethanol triggers both hepatic and cardiac sympathetic activation, leading to steatohepatitis with advanced fibrosis and cardiac dysfunction in the absence of obesity, hyperlipidemia, or hyperglycemia. This suggests that sympathetic activation plays a role in the development of these conditions. Novel strategies to treat patients with non-obese MASLD with cardiac dysfunction may aim at resetting hepatic inflammation and myocardial fibrosis through the suppression of sympathetic activity.

## 5. Conclusions

This study describes a non-obese MASLD mouse model that develops steatohepatitis with advanced liver fibrosis. This was achieved by feeding 7-week-old male mice a combined diet of STHD-01 and ethanol for 12 weeks. This model successfully replicates key features of the human condition in a controlled experimental setting. Myocardial fibrosis-associated cardiac dysfunction, and cardiac and hepatic sympathetic activation are all observed in the present mouse model. Our findings might contribute to novel treatment strategies for advanced non-obese MASLD with cardiac dysfunction.

## Data Availability

The data generated or analyzed during this study are provided in this published article.

## References

[1] Younossi Z., Anstee Q.M., Marietti M., Hardy T., Henry L., Eslam M., George J., Bugianesi E. (2018). Global burden of NAFLD and NASH: Trends, predictions, risk factors and prevention. Nat. Rev. Gastroenterol. Hepatol..

[2] Sarin S.K., Kumar M., Eslam M., George J., Al Mahtab M., Akbar S.M.F., Jia J., Tian Q., Aggarwal R., Muljono D.H. (2020). Liver diseases in the Asia-Pacific region: A Lancet Gastroenterology & hepatology Commission. Lancet Gastroenterol. Hepatol..

[3] Ahadi M., Molooghi K., Masoudifar N., Namdar A.B., Vossoughinia H., Farzanehfar M. (2021). A review of non-alcoholic fatty liver disease in non-obese and lean individuals. J. Gastroenterol. Hepatol..

[4] Younossi Z.M., Stepanova M., Negro F., Hallaji S., Younossi Y., Lam B., Srishord M. (2012). Nonalcoholic fatty liver disease in lean individuals in the United States. Medicine.

[5] Fan J.G., Kim S.U., Wong V.W. (2017). New trends on obesity and NAFLD in Asia. J. Hepatol..

[6] Ye Q., Zou B., Yeo Y.H., Li J., Huang D.Q., Wu Y., Yang H., Liu C., Kam L.Y., Tan X.X.E. (2020). Global prevalence, incidence, and outcomes of non-obese or lean non-alcoholic fatty liver disease: A systematic review and meta-analysis. Lancet Gastroenterol. Hepatol..

[7] Lu F.B., Zheng K.I., Rios R.S., Targher G., Byrne C.D., Zheng M.H. (2020). Global Epidemiology of Lean Non-Alcoholic Fatty Liver Disease: A Systematic Review and Meta-Analysis. J. Gastroenterol. Hepatol..

[8] Leung J.C.F., Loong T.C.W., Wei J.L., Wong G.L.H., Chan A.W.H., Choi P.C.L., Shu S.S.T., Chim A.M.L., Chan H.L.Y., Wong V.W.S. (2017). Histological severity and clinical outcomes of nonalcoholic fatty liver disease in nonobese patients. Hepatology.

[9] Donnelly K.L., Smith C.I., Schwarzenberg S.J., Jessurun J., Boldt M.D., Parks E.J. (2005). Sources of fatty acids stored in liver and secreted via lipoproteins in patients with nonalcoholic fatty liver disease. J. Clin. Investig..

[10] Bugianesi E., Gastaldelli A., Vanni E., Gambino R., Cassader M., Baldi S., Ponti V., Pagano G., Ferrannini E., Rizzetto M. (2005). Insulin resistance in non-diabetic patients with non-alcoholic fatty liver disease: Sites and mechanisms. Diabetologia.

[11] Valbusa F., Agnoletti D., Scala L., Grillo C., Arduini P., Bonapace S., Calabria S., Scaturro G., Mantovani A., Zoppini G. (2018). Non-alcoholic fatty liver disease and increased risk of all-cause mortality in elderly patients admitted for acute heart failure. Int. J. Cardiol..

[12] Nucera S., Ruga S., Cardamone A., Coppoletta A.R., Guarnieri L., Zito M.C., Bosco F., Macrì R., Scarano F., Scicchitano M. (2022). MAFLD progression contributes to altered thalamus metabolism and brain structure. Sci. Rep..

[13] Tsutsumi T., Eslam M., Kawaguchi T., Yamamura S., Kawaguchi A., Nakano D., Koseki M., Yoshinaga S., Takahashi H., Anzai K. (2021). MAFLD better predicts the progression of atherosclerotic cardiovascular risk than NAFLD: Generalized estimating equation approach. Hepatol. Res..

[14] Adams L.A., Lymp J.F., Sauver J.S., Sanderson S.O., Lindor K.D., Feldstein A., Angulo P. (2005). The natural history of nonalcoholic fatty liver disease: A population-based cohort study. Gastroenterology.

[15] Ahmed O.T., Gidener T., Mara K.C., Larson J.J., Therneau T.M., Allen A.M. (2022). Natural history of nonalcoholic fatty liver disease with normal body mass index: A population-based study. Clin. Gastroenterol. Hepatol..

[16] Golabi P., Paik J., Fukui N., Locklear C.T., de Avilla L., Younossi Z.M. (2019). Patients with lean nonalcoholic fatty liver disease are metabolically abnormal and have a higher risk for mortality. Clin. Diabetes Publ. Am. Diabetes Assoc..

[17] McDonagh T.A., Metra M., Adamo M., Gardner R.S., Baumbach A., Bohm M., Burri H., Butler J., Celutkiene J., Chinocel O. (2021). 2021 ESC Guidelines for the diagnosis and treatment of acute and chronic heart failure. Eur. Heart J..

[18] Van Riet E.E., Hoes A.W., Wagenaar K.P., Limburg A., Landman M.A., Rutten F.H. (2016). Epidemiology of heart failure: The prevalence of heart failure and ventricular dysfunction in older adults over time. A systematic review. Eur. J. Heart Fail..

[19] Boyle M., Masson S., Anstee Q.M. (2018). The bidirectional impacts of alcohol consumption and the metabolic syndrome: Cofactors for progressive fatty liver disease. J. Hepatol..

[20] Oben J.A., Roskams T., Yang S., Lin H., Sinelli N., Li Z., Torbenson M., Thomas S.A., Diehl A.M. (2003). Norepinephrine induces hepatic fibrogenesis in leptin deficient ob/ob mice. Biochem. Biophys. Res. Commun..

[21] Oben J.A., Diehl A.M. (2004). Sympathetic nervous system regulation of liver repair. Anat. Rec. A Discov. Mol. Cell. Evol. Biol..

[22] Liu J., Fujimiya T. (2010). Abrupt termination of an ethanol regimen provokes ventricular arrhythmia and enhances susceptibility to the arrhythmogenic effects of epinephrine in rats. Alcohol Clin. Exp. Res..

[23] Liu J., Takase I., Hakucho A., Okamura N., Fujimiya T. (2012). Carvedilol attenuates the progression of alcohol fatty liver disease in rats. Alcohol Clin. Exp. Res..

[24] Liu J. (2020). Alcohol consumption combined with dietary low-carbohydrate/high-protein intake increased the left ventricular systolic dysfunction risk and lethal ventricular arrhythmia susceptibility in apolipoprotein E/low-density lipoprotein receptor double-knockout mice. Alcohol.

[25] Ejima C., Kuroda H., Ishizaki S. (2016). A novel diet-induced murine model of steatohepatitis with fibrosis for screening and evaluation of drug candidates for nonalcoholic steatohepatitis. Physiol. Rep..

[26] Cheitlin M.D., Armstrong W.F., Aurigemma G.P., Beller G.A., Bierman F.Z., Davis J.L., Douglas P.S., Faxon D.P., Gillam L.D., Kimball T.R. (2003). ACC/AHA/ASE 2003 guideline update for the clinical application of echocardiography: Summary article. A report of the American College of Cardiology/American Heart Association Task Force on Practice Guidelines (ACC/AHA/ASE Committee to Update the 1997 Guidelines for the Clinical Application of Echocardiography). J. Am. Soc. Echocardiogr..

[27] Furuta Y., Liu J., Himemiya-Hakucho A., Yoshimura K., Fujimiya T. (2019). Alcohol Consumption in Combination with an Atherogenic Diet Increased Indices of Atherosclerosis in Apolipoprotein E/Low-Density Lipoprotein Receptor Double-Knockout Mice. Alcohol Clin. Exp. Res..

[28] Gadd V.L., Skoien R., Powell E.E., Fagan K.J., Winterford C., Horsfall L., Irvine K., Clouston A.D. (2014). The portal inflammatory infiltrate and ductular reaction in human nonalcoholic fatty liver disease. Hepatology.

[29] Erlinger S. (2011). Do patients with nonalcoholic fatty liver disease die from their heart?. Clin. Res. Hepatol. Gastroenterol..

[30] Sheka A.C., Adeyi O., Thompson J., Hameed B., Crawford P.A., Ikramuddin S. (2020). Nonalcoholic Steatohepatitis: A Review. JAMA.

[31] Liang W., Menke A.L., Driessen A., Koek G.H., Lindeman J.H., Stoop R., Havekes L.M., Kleemann R., van den Hoek A.M. (2014). Establishment of a general NAFLD scoring system for rodent models and comparison to human liver pathology. PLoS ONE.

[32] Liu J., Oba Y., Seiko Yamano S. (2022). Chronic ethanol consumption plus dietary atherogenic diet intake created metabolic steatohepatitis with advanced liver fibrosis in apolipoprotein E/low-density lipoprotein receptor double-knockout mice. Alcohol Clin. Exp. Res..

[33] Rice B.A., Naimi T.S., Long M.T. (2022). Nonheavy alcohol use associates with liver fibrosis and nonalcoholic steatohepatitis in the Framingham Heart Study. Clin. Gastroenterol. Hepatol..

[34] Long M.T., Massaro J.M., Hoffmann U., Benjamin E.J., Naimi T.S. (2020). Alcohol use is associated with hepatic steatosis among persons with presumed nonalcoholic fatty liver disease. Clin. Gastroenterol. Hepatol..

[35] Weiskirchen R., Weiskirchen S., Tacke F. (2018). Recent advances in understanding liver fibrosis: Bridging basic science and individualized treatment concepts. F1000Res.

[36] Adams L.A. (2019). End-points for drug treatment in NASH. Hepatol. Int..

[37] Janig W. (2014). Sympathetic nervous system and inflammation: A conceptual view. Auton. Neurosci..

[38] Cervi A.L., Lukewich M.K., Lomax A.E. (2014). Neural regulation of gastrointestinal inflammation: Role of the sympathetic nervous system. Auton. Neurosci..

[39] Wang Y., Jiang W., Chen H., Zhou H., Liu Z., Liu Z., Liu Z., Zhou Y., Zhou X., Yu L. (2021). Sympathetic nervous system mediates cardiac remodeling after myocardial infarction in a circadian disruption model. Front. Cardiovasc. Med..

